# Artificial intelligence improves the accuracy of residents in the diagnosis of hip fractures: a multicenter study

**DOI:** 10.1186/s12891-021-04260-2

**Published:** 2021-05-03

**Authors:** Yoichi Sato, Yasuhiko Takegami, Takamune Asamoto, Yutaro Ono, Tsugeno Hidetoshi, Ryosuke Goto, Akira Kitamura, Seiwa Honda

**Affiliations:** 1 Department of Orthopedics Surgery, Gamagori City Hospital, Gamagori, Japan; 2 Nonprofit Organization (NPO) Nagoya Orthopedic Regional Healthcare Support Center, AI Research Division, Meitohonmachi 2-22-1, Meito-ward, Nagoya, Japan; 3 Department of Orthopedic Surgery, Nagoya University Graduate School of Medicine, Nagoya, Japan; 4 Department of Orthopedics Surgery, Tsushima City Hospital, Thushima, Japan; 5 Department of Orthopedics Surgery, Nagoya Daini Red Cross Hospital, Nagoya, Japan; 6 Search Space CO,Ltd., Hatagaya 3-39-12, Shibuya-ward, Tokyo, Japan

## Abstract

**Background:**

Less experienced clinicians sometimes make misdiagnosis of hip fractures. We developed computer-aided diagnosis (CAD) system for hip fractures on plain X-rays using a deep learning model trained on a large dataset. In this study, we examined whether the accuracy of the diagnosis of hip fracture of the residents could be improved by using this system.

**Methods:**

A deep convolutional neural network approach was used for machine learning. Pytorch 1.3 and Fast.ai 1.0 were applied as frameworks, and an EfficientNet-B4 model (a pre-trained ImageNet model) was used. We handled the 5295 X-rays from the patients with femoral neck fracture or femoral trochanteric fracture from 2009 to 2019. We excluded cases in which the bilateral hips were not included within an image range, and cases of femoral shaft fracture and periprosthetic fracture. Finally, we included 5242 AP pelvic X-rays from 4851 cases. We divided these 5242 images into two images per image, and prepared 5242 images including fracture site and 5242 images without fracture site. Thus, a total of 10,484 images were used for machine learning. The accuracy, sensitivity, specificity, F-value, and area under the curve (AUC) were assessed. Gradient-weighted class activation mapping (Grad-CAM) was used to conceptualize the basis for the diagnosis of the fracture by the deep learning algorithm. Secondly, we conducted a controlled experiment with clinicians. Thirty-one residents;young doctors within 2 years of graduation from medical school who rotate through various specialties, were tested using 300 hip fracture images that were randomly extracted from the dataset. We evaluated the diagnostic accuracy with and without the use of the CAD system for each of the 300 images.

**Results:**

The accuracy, sensitivity, specificity, F-value, and AUC were 96.1, 95.2, 96.9%, 0.961, and 0.99, respectively, with the correct diagnostic basis generated by Grad-CAM. In the controlled experiment, the diagnostic accuracy of the residents significantly improved when they used the CAD system.

**Conclusions:**

We developed a newly CAD system with a deep learning algorithm from a relatively large dataset from multiple institutions. Our system achieved high diagnostic performance. Our system improved the diagnostic accuracy of residents for hip fractures.

**Level of evidence:**

Level III, Foundational evidence, before-after study. Clinical relevance: high

**Supplementary Information:**

The online version contains supplementary material available at 10.1186/s12891-021-04260-2.

## Background

In Japan, as many as 13 million elderly people have osteoporosis [1, 2]. Fragility fractures, such as hip fractures and spinal fractures are also increasing, with 200,000 patients suffering from hip fractures annually [3]. Patients with hip fractures require admission to hospital as soon as possible, because the longer the patients delay getting treatment, the worse their walking ability and prognosis will be [4, 5].

Most hip fracture patients visit the emergency department because they have difficulty walking due to pain. In the emergency department, clinicians are exposed to excessive time and mental stress, which can cause fatigue and misdiagnosis [6, 7]. This tendency is particularly pronounced among residents [8]. In previous studies, the misdiagnosis rate at the initial diagnosis for hip fractures was estimated to be 2–10% [9].

A delay in the diagnosis and treatment worsens the prognosis [10], and a misdiagnosis may lead to medical litigation [6]. To prevent a misdiagnosis, radionuclide bone scans, computed tomography (CT), and magnetic resonance imaging (MRI), as well as plain X-rays, are recommended as additional diagnostic imaging [11, 12]. However, these additional tests are not always available in all institutions.

In recent years, deep learning, a method of machine learning using multi-layered neural networks, has emerged and improved the accuracy of image recognition [13]. In the field of medicine, many previous studies have reported the application of deep learning to imaging analysis and demonstrated its high diagnostic accuracy [14]. Several studies have applied a deep learning algorithm to the diagnosis of fractures [15]. Olczak first demonstrated that artificial intelligence (AI) with the use of a deep learning approach for the diagnosis of ankle and wrist fracture on plain X-rays [16]. There have been some papers on the use of a deep learning algorithm to diagnose hip fractures. Some of these studies diagnose from antero-posterior images only [17–20], one from both antero-posterior and lateral images [21], one can predict not only presence of fractures but also fracture type [22], and one of the same algorithms can be used to diagnose both proximal femur and pelvic fractures [23] . Additionally, a previous study reported that deep learning algorithm improved the diagnostic accuracy of fracture detection by clinicians [20, 22, 24]. However, these studies were conducted in a single center. The dataset was relatively small and the image processing method was uniform. Few studies have described the improvement of clinicians’ diagnostic accuracy for hip fractures with the aid of deep learning algorithms and no studies have reported differences in outcomes according to years of clinical experience.

Thus, we planned to train a deep learning model using a large dataset with images obtained by various protocols in a multi-institutional setting. We newly developed the computer-aided diagnosis (CAD) system using a model that could visualize the diagnostic method of the AI. In the present study, we hypothesized that the CAD system would improve the diagnostic accuracy of clinicians, including residents.

## Methods

### Subjects

All research contents were performed in accordance with the ethical standards of the amended Declaration of Helsinki. This study was conducted with the approval of the ethics committee of each hospital (Gamagori City Hospital: approval No. 368–1, Tsushima City Hospital: Approval No. 2019–3, Nagoya Daini Red Cross Hospital: approval No. 1360).

We collected images from 3 hospitals (Gamagori City Hospital, Tsushima City Hospital, Nagoya Daini Red Cross Hospital) in Aichi Prefecture, Japan. The Nagoya Daini Red Cross Hospital provided tertiary care in an urban area with a population of 2.3 million. The other two hospitals—Gamagori City Hospital and Tsushima City Hospital—are primary care hospitals in a rural area in Japan. Table 1 shows the background factors of each institution.

**Table 1:** Information about the participating medical institutions

	Gamagori City Hospital	Tsushima City Hospital	Nagoya Daini Red Cross Hospital	Overall	*P*-value
Medical sphere (Number of people)	140,000	300,000	570,000	1,010,000	< 0.001*
Number of ambulances per year in 2019	3351	4380	12,726	20,457	< 0.001*
Number of emergency patients in 2019	14,131	13,724	37,713	65,568	< 0.001*
Number of residents in 2019	7	11	47	65	< 0.001*
X-ray generator	MODEL TF-6TL-6 (TOSHIBA, Japan)	UD150L-40 (SHIMADZU, Japan)	DHF-153HII (HITACHI, Japan)		
Image processing unit	CALNEO Smart C12 (FUJIFILM, Japan)	Aero DR (Konica Minolta, Japan)	FCR VELOCITY (FUJITSU, Japan)		
Image file format	.jpeg	.dcm	.jpeg		
Image size	4892 × 4020 pixel	3451 × 2836 pixel	2039 × 1380 pixel		

* *P* < 0.05

**Fig. 1:**
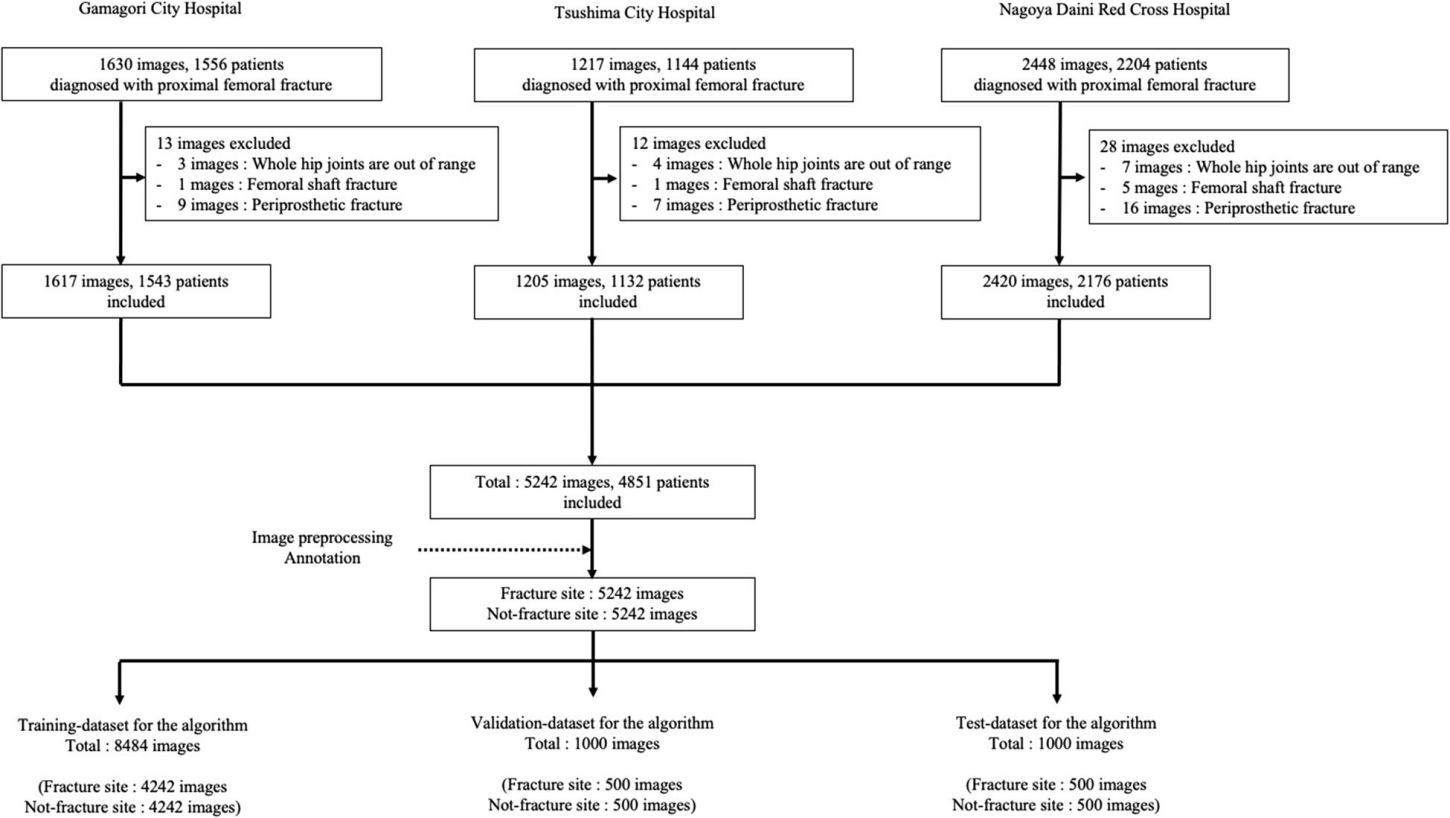
Patient flow

We collected 5295 cases of femoral neck fractures or femoral trochanteric fractures that were diagnosed by orthopedic surgeons using plain X-ray, CT or MRI between 2009 and 2019. Patients aged 20 years and older were included in the study. Among these 5295 cases, 391 cases subsequently suffered a hip fracture on the opposite side during the study period. We also included hip implants on the opposite side (*n* = 452), complicated pubic or sciatic fracture (*n* = 93), cases with osteoarthritis of the hip (Kellgren–Lawrence Grade III or IV: 84 cases) [25], including spine implants (*n* = 46), and pathologic fractures of the proximal femur due to metastatic cancer (*n* = 12). We excluded images for the following reasons: periprosthetic fractures (*n* = 32), bilateral hips were not included within an image range (*n* = 14), and femoral shaft fracture (*n* = 7). Finally, we utilized 5242 AP pelvic X-rays in 4851 cases (Sex: male, *n* = 1193; female, *n* = 3658, mean age at injury: 81.1 years) (Fig. 1). Of these, we diagnosed 5024 (95.8%) from frontal simple hip radiographs, 97 (1.9%) with radiographic lateral views as well, and 121 (2.3%) with CT or MRI for definitive or exclusionary diagnosis.

### Evaluation of fractures

Two orthopedic surgeons (YS, TA) assessed the presence or absence and the type of fracture. The Kappa statistic of inter-observer correlation for the presence or absence of these fractures was 0.91. If the results differed, it was decided after a discussion. To classify the fracture type, we used the Garden classification (Garden classification stage I, II, III, IV: G/S I-IV) for femoral neck fractures [26] and the AO/OTA classification for femoral trochanteric fractures (AO/OTA 31-A1, A2, A3) [27]. We defined a great trochanteric fracture as one in which the fracture line did not extend to the medial cortex [28]. A total of 5024 cases (95.8%) were diagnosed from AP pelvic X-rays alone. Other patients were diagnosed by lateral X-ray (*n* = 97; 1.9%) and CT or MRI (*n* = 121; 2.3%). All of these images were incorporated into the dataset without any specific labeling, and were being trained equally. Table 2 shows the classifications of fracture types.

**Table 2:** Patient background and the classification of fracture type

		Gamagori City Hospital	Tsushima City Hospital	Nagoya Daini Red Cross Hospital	Overall	*P*-value
Mean age at time of injury (95% CI)		81.8 (70.4, 93.2)	81.4 (70.9, 91.9)	80.1 (67.6, 92.6)	81.1 (69.6, 92.6)	< 0.001*
Sex (male/female)		340/1156	287/829	566/1673	1193/3658	0.13
Fracture type	Garden (I,II/III,IV)	275/450	191/324	528/897	994/1671	< 0.001*
	AO31- (A1/A2/A3)	489/253/54	383/185/48	509/322/76	1381/760/178	0.09
	Greater trochanteric fracture	96	74	88	258	< 0.001*
Number of X-rays		1617	1205	2420	5242	
Complications	Pathologic fractures due to tumors	3	2	7	12	0.80
	Osteoarthritis of the hip	26	15	43	84	0.50
	Hip implants on the opposite side	132	125	195	452	0.05
	Spine implants	7	4	35	46	< 0.001*
	Complicated pubic or sciatic fracture	23	17	53	93	0.12

Age at injury and fracture type were evaluated for each fracture site when there were multiple images in bilateral cases

*95%CI* 95% confident intervals

* *P* < 0.05

### Image capturing environment and image data extraction

X-rays were taken with the patient in the supine position with internal rotation of their hip. The central beam in the AP view was directed to the midpoint between the upper border of the pubic symphysis and a horizontal line connecting both anterior iliac spines. The imaging conditions were as follows: 70 kV, 200 mA, 0.4 s, and tube-to-film distance, 100 cm. The X-ray generator and CR or DR image processor. We used a Digital Imaging and Communications in Medicine (DICOM) image display (Toshiba Medical Systems Corporation, Tokyo) as the image reference software program. The image file format and the size of the original image varied from each institution. (Table 1).

### Image preprocessing and development of the algorithm

We described the details image processing and the algorithm in Supplemental method. We used uncompressed data. Images extracted from the DICOM server were converted into 3 channels and 8-bit JPEG images, and both were resized to 380 × 380 pixels. For each image, the window level was not adjusted. All images were given a rectangle that included the entire fracture site. To extract larger images, we placed a vertical dividing line at a position with a 50-pixel margin for the rectangle and adopted the images without the rectangle as the non-fractured side data and generated 5242 images that did not contain the fracture site. We also adopted the image of the side containing the rectangle of the same size as the non-fractured site data as the fracture side data and generated 5242 images containing the fracture site. In total, 10,484 images were prepared for machine learning (Supplementary Figure 1).

We randomly divided the dataset into three sets: a training dataset (non-fracture sides, *n* = 4242; fracture side, *n* = 4242; total, 8484 images), a validation dataset (non-fracture side, *n* = 500; fracture side, *n* = 500; for a total of 1000 images), and a test dataset (non-fracture side, *n* = 500; fracture side, *n* = 500; for a total of 1000 images) (Fig. 1).

We used gradient-weighted class activation mapping (Grad-CAM) [29] to conceptualize the basis for the deep learning algorithm’s diagnosis of a fracture. We used the show-heatmap-function of Fast.AI (http://www.fast.ai) on the deep learning algorithm to obtain the heatmap. Through this process, we have developed a CAD system based on a deep learning algorithm that provides diagnosis and visualization of basis.

We determined the calculation time for the whole process of the inference and the generation of heat maps for one image of the test dataset. The calculation method is the average time per image of the test dataset divided by the calculation time, which was deduced from 1000 images of test data.

### Controlled experiment with clinicians

To investigate the application of the CAD system and verify its effectiveness in a clinical setting, we conducted a controlled experiment with clinicians. There were 65 residents;young doctors within 2 years of graduation from medical school without speciality, in the three institutions included in the study. Thirty-one of these residents agreed to participate in the study (10 in their first year of residency, and 21 in their second year of residency). Each of these participants provided their informed consent at their respective institutions.

We randomly extracted 300 images (133 on the non-fractured side and 167 on the fractured side) from 1000 test image datasets described as a previous study [24]. The 300 images included 136 right femur images and 164 left femur images. First, we checked the performance of the deep learning algorithm for the 300 images.

Then, clinicians undertook the diagnostic test. Before conducting the test, we presented the accuracy of the CAD to the clinicians. The outline of the diagnostic test was as follows: 1) the clinicians diagnosed the presence or absence of fracture by themselves; 2) after the clinician answered, the CAD system added the visualization of the fracture to the same image; 3) as a second test, the clinician responded again based on the hint. (Supplemental Figure 4) This sequence was repeated 300 times.

### Assessment

#### Performance of the deep learning algorithm

We evaluated the performance of the trained deep learning algorithm using the test image dataset. We also calculated the accuracy, sensitivity, specificity, F-value, receiver operating characteristic (ROC) curve and measured the area under the curve (AUC), as described in the STARD 2015 guidelines [30].

#### Evaluation of the heatmap generated by the CAD system

We performed accuracy validation of Grad-CAM in accordance with the previous research [18]. We used a total of 40 images, 20 with and 20 without fractures, randomly selected from images that the algorithm was able to correctly diagnose in the test data set, for accuracy validation. For accuracy validation, we used the area with the highest signal intensity in the Heat map as the basis for determining “with fracture” if it was located directly above the femur between the femoral head and just above the popliteus. The assessor (YS) evaluated the consistency between the high signal intensity region on the heat map and the actual fracture site on the X-ray using sensitivity and specificity. The kappa value for intra-observer correlation between two-week intervals was 1.0.

#### The diagnostic accuracy of clinicians with or without the use of the CAD system

We compared the accuracy, sensitivity, and specificity with/without the aid of the CAD system among residents. We also compared the diagnostic accuracy of the first-year residents to that of second-year residents.

### Statistical analysis

The EZR software program was used to perform the statistical analyses [16]. We used Fisher’s exact test were used to analyze categorical variables. The normality of the distribution of diagnostic accuracy was tested for using the Shapiro-Wilk test. As a result, the value did not show a normal distribution. Thus, we used the Wilcoxon signed-rank test. *P* values of < 0.05 were considered to indicate statistical significance. Scikit-Learn (https://scikit-learn.org/) was used to analyze the performance of the deep learning algorithm.

## Results

### Performance of the deep learning algorithm

**Fig. 2:**
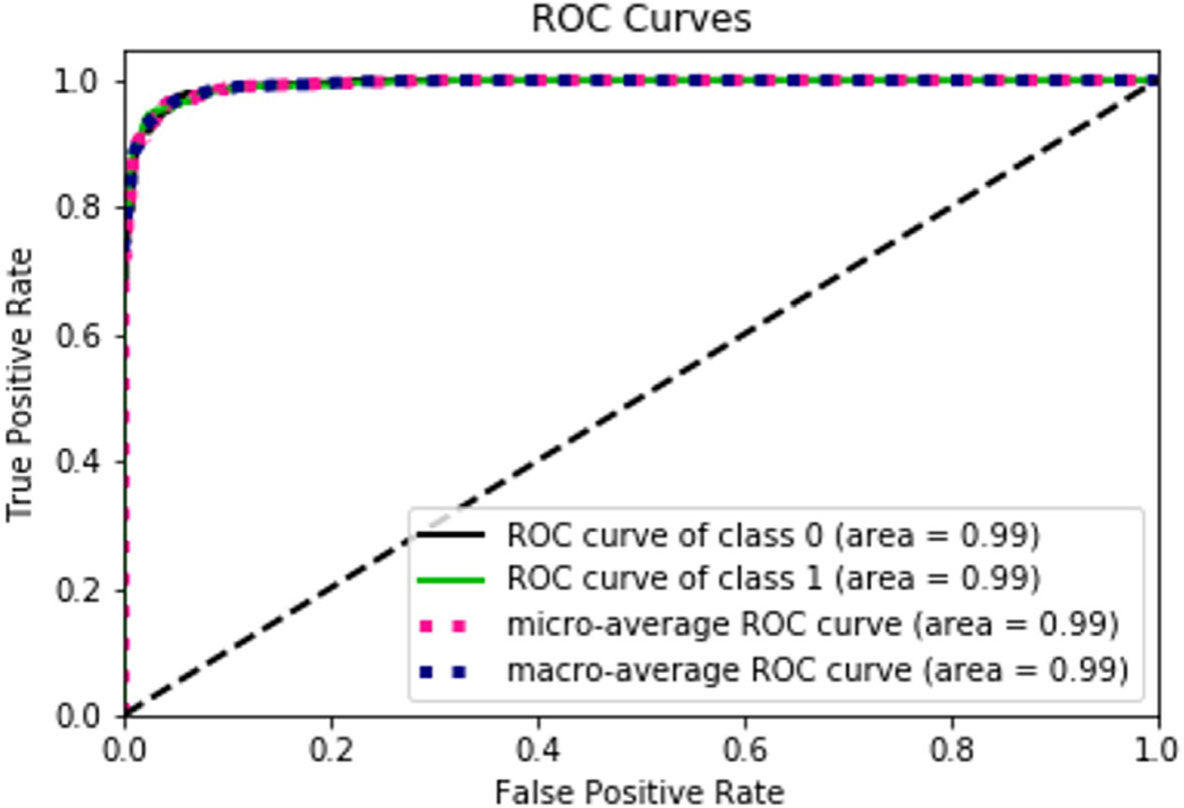
ROC Curves. This is the ROC curve for the EfficientNet-B4 model, which showed an AUC of 0.992. Class 0 indicates cases without fracture, and Class 1 indicates cases with fracture. Each ROC curve was calculated. The micro-average ROC sums contributions by class, while the macro-average ROC shows the average results for all classes (AUC = 0.992)

The performance of the deep learning algorithm was as follows: accuracy, 96.1% (95% CI: 94.9, 97.3); sensitivity, 95.2% (95% CI: 93.9, 96.5); specificity, 96.9% (95% CI: 95.8, 98.0), and F-value, 0.961 (95% CI: 0.950, 0.972). The ROC curve is shown in Fig. 2; the AUC was 0.99 (95% CI, 0.98, 1.00).

**Fig. 3:**
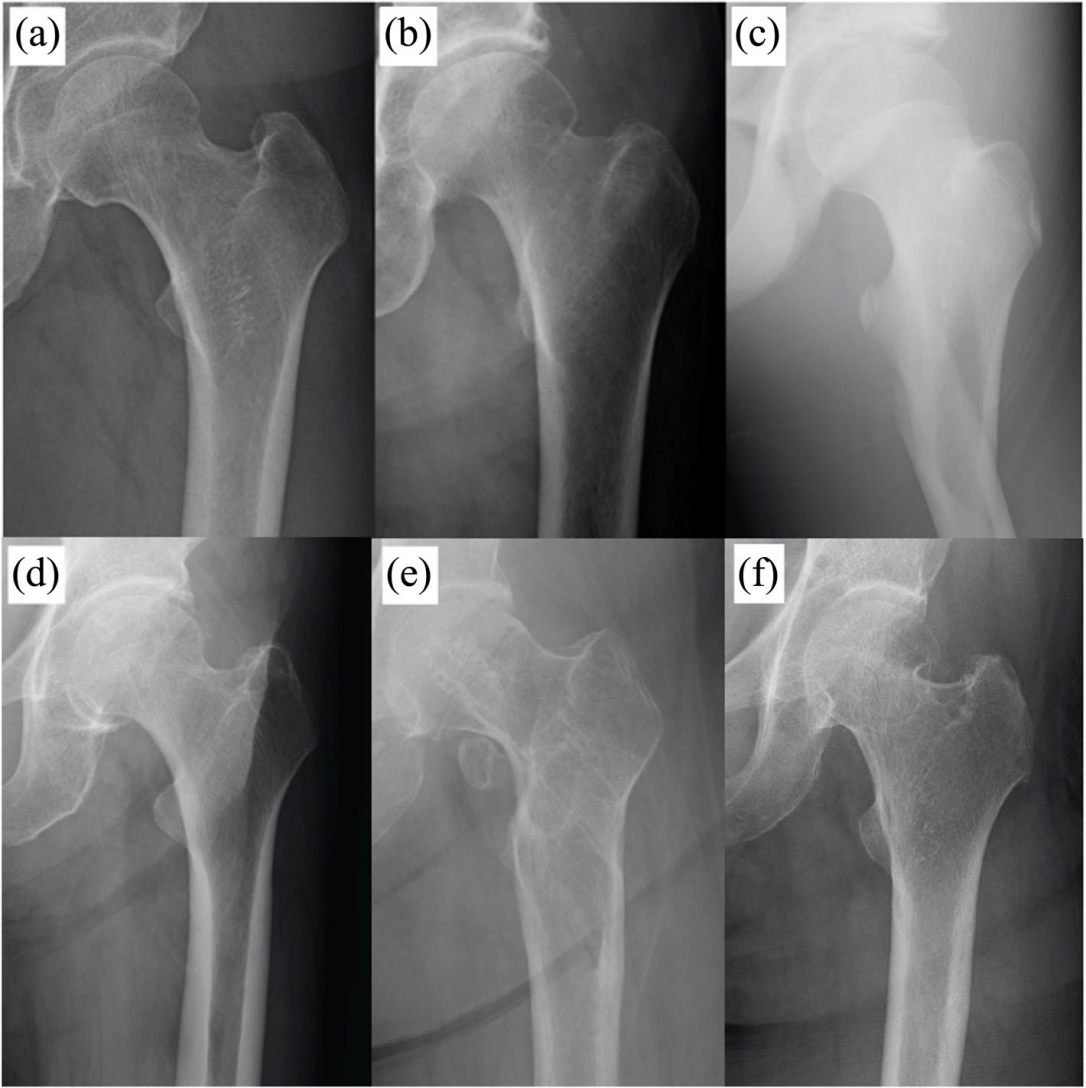
Images that were misdiagnosed by the CAD system. **a**-**c** Incorrectly diagnosed by the CAD system (false-negative). **a** A case that even orthopedic surgeons could not decide. **b** A case in which a non-orthopedic surgeon could be wrong. **c** A case in which even non-orthopedic surgeons were not confused by the diagnosis. **d-f** Images that were incorrectly diagnosed by the CAD system (false-positive). **d** Normal image. **e** A case after implant removal. **f** A case in which deformity healed after conservative treatment

On the other hand, the deep learning algorithm misdiagnosed 39 of the 1000 images. A total of 24 images with fractures were diagnosed as “without fracture” (false negative). These included slightly displaced fractures (*n* = 21); fractures located at the greater trochanter of the femur (*n* = 9), non-displaced femoral neck fractures (*n* = 8), femoral trochanteric fractures (*n* = 8; AO31 - A1). The others included relatively displaced fractures (*n* = 3); femoral trochanteric fracture (*n* = 2; AO31 - A2, 3), and displaced femoral neck fracture (*n* = 1; G/S 3, 4). A total of 15 images without fracture were diagnosed as “with fracture” (false-positive). These included 13 cases with normal images, a case with deformity after conservative treatment and a case after nail removal (Fig. 3).

### Evaluation of the heatmap generated by the CAD system

**Fig. 4:**
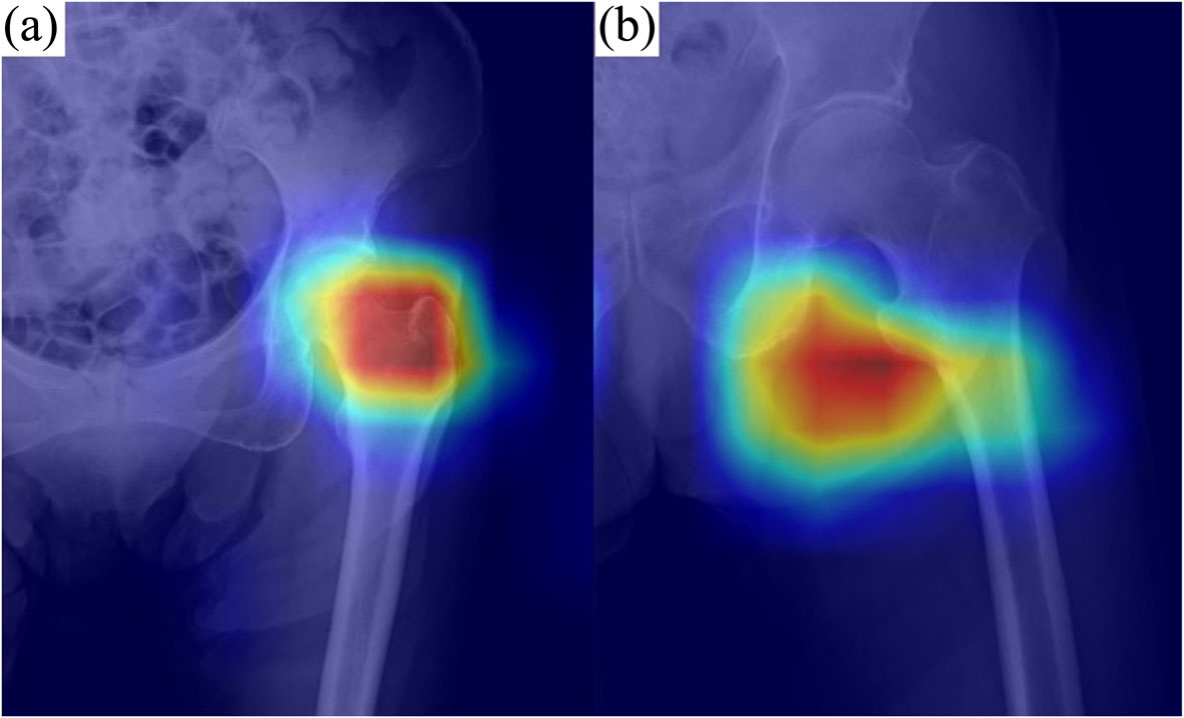
Visualization of the area of fracture detection using Grad-CAM. **a** For images diagnosed by the algorithm as “with fracture”, Grad-CAM showed a high-signal region consistent with the fracture site . **b** For images diagnosed as “no fracture”, Grad-CAM showed high-signal areas in the region other than femoral neck and trochanteric. From red to green, the diagnostic basis of the CAD system was strongly evident

For images diagnosed by the algorithm as “with fracture”, Grad-CAM showed a high-signal region consistent with the fracture site. For images diagnosed as “without fracture”, Grad-CAM showed high-signal areas in the region other than femoral neck and trochanteric. (Fig. 4) For the 20 “with Fracture” images, all 20 images had the same high-signal region on the heat map as the fracture site. On the other hand, 19 of the 20 “without fracture” images had high signal areas except from the femoral head to just above the trochanter in the 19 images, but one image had a high signal area in the greater trochanter. (Supplemental Figure 5) The accuracy of Grad-CAM was thus calculated to be 100% sensitivity and 95% specificity. The average inference time per image, including Grad-CAM, was 1.17 s.

### The diagnostic accuracy of clinicians with or without the aid of the CAD system

The residents’ mean diagnostic accuracy was significantly improved with the aid of the CAD system. (accuracy of 84.7% (95% CI: 82.2, 87.2) without aid to 91.2% (95% CI: 89.6, 92.8) with aid; *p* < 0.01, sensitivity of 83.4% (95% CI: 83.4, 90.6) with aid to 90.6% (95% CI: 83.9, 97.3) with aid *p* < 0.01, specificity of 88.7% (95% CI: 82.1, 95.3) without aid to 93.4% (95% CI: 89.5, 97.3) with ai *p* < 0.01).

The results of the diagnostic accuracy of residents in both the first and second years are presented in Table 3. The accuracy, sensitivity, and specificity of the residents were improved with the CAD system, irrespective of their year of residency.

**Table 3:** Results of the controlled experiment in which clinicians diagnosed 300 test images

	Accuracy (%)			Sensitivity (%)			Specificity (%)		
	With CAD	Without CAD	*P* value	With CAD	Without CAD	*P* value	With CAD	Without CAD	*P* value
First-year residents (95%CI)	82.1 (78.6, 85.7)	91.2 (89.3, 93.0)	< 0.001*	81.9 (79.4, 84.3)	90.5 (88.5, 92.5)	< 0.001*	86.5 (83.0, 90.1)	93.2 (91.3, 95.1)	0.08
Second-year residents (95%CI)	85.9 (83.9,87.9)	90.9 (89.3, 92.6)	< 0.001*	84.2 (81.4, 87.0)	90.6 (88.0, 93.2)	< 0.001*	89.7 (88.2, 91.2)	93.5 (92.4, 94.7)	< 0.001*

*95%CI* 95% confident intervals

* *P* < 0.05

## Discussion

We developed a newly CAD system based on a deep learning algorithm for hip fracture. This system provided high accuracy, sensitivity, and specificity. The areas activated on the heat map all corresponded to the areas pointed out by the orthopedic surgeon. Inexperienced residents’ diagnostic accuracy, sensitivity, and specificity in the diagnosis of hip fracture improved when they used the CAD system.

Our CAD system, based on a deep learning algorithm, had some advantages over other studies. We conducted a literature review that demonstrated the application of AI-based systems for the diagnosis of hip fracture in Table 4 [17–22]. We used the largest amount of learning data from multiple institutions. In this study, almost all of the images of hip fractures obtained from multiple institutions were used, and approximately 10,000 images of machine learning data were generated from approximately 5000 cases. Large datasets are the key to success in machine learning [31]. The majority of published studies on AI to date were conducted in a single institution; only 6% of these studies used data from multiple institutions [32]. Our multiple-center dataset provides 1) a large amount of data, and 2) images with different imaging formats. In this study, the deep learning algorithm achieved high accuracy at multiple institutions, despite the use of different radiographic equipment and image file formats. The high performance of the multi-center data may help in the practical application of this system.

**Table 4:** Literature review

	Year	Insti-tution	Number of patients	Number of images for machine learning	Fracture type (femoral neck/ trochanteric fracture)	Images including implants on hip or spine	Accuracy (%)	Sensitivity (%)	Specificity (%)	AUC	Grad-CAM	Clinician test (AI-aided test)
Adams et al. [17]	2018	1	805	805	femoral neck fracture	excluded	90.6	N/A	N/A	0.98	no	no
Urakawa et al. [19]	2018	1	1773	3346	femoral trochanteric fracture	excluded	95.5	93.9	97.4	0.97	no	no
Cheng et al. [18]	2019	1	3605	3605	both	included	91	98	84	0.98	yes	no
Yamada et al. [21]	2019	1	1047	2923	both	excluded	98.0	98.0	98.0	N/A	no	no
Krogue et al. [22]	2020	1	1118	3026	both	included	93.7	93.2	94.2	0.98	yes	yes
Cheng et al. [20]	2020	1	3605	3605	both	excluded	91.0	98.0	84.0	N/A	yes	yes
Current study	2021	3	4851	10,484	both	included	96.1	95.2	96.9	0.99	yes	yes

The performance of our deep learning algorithm was as good as that described in previous reports. On the other hand, the deep learning algorithm failed to diagnose 3.9% of images (39 out of 1000 test data) correctly. Twenty-four images with fractures were diagnosed as “without fracture” and 15 images without fracture were diagnosed as “with fracture”.

Second, our CAD system, which was based on a deep learning algorithm, was able to provide a heat map of the fracture site, which provided evidence about where the AI recognized the fracture. In all cases, the fracture site indicated on the heat map was located in the area indicated by the orthopedic surgeon. AI-based diagnostics has classically been associated with a “black box problem” [33], in that cannot explicitly express the feature quantity, the reasons for the judgment are not clear, and humans cannot understand or interpret the reasons. In this study, we used Grad-CAM to visualize class-discriminative regions on the X-rays. This could reveal the location of the diagnosis. However, the Grad-CAM could show the fracture as a rough area, but cannot show the fracture line itself. Besides, the image information that the deep learning algorithm based its decision on (e.g., the fracture line, bone marrow edema, or soft-tissue contrast) is still unclear.

Third, in this study, the diagnostic accuracy, sensitivity, and specificity of residents improved when they used the CAD system. Moreover, the CAD system improved their diagnostic accuracy regardless of the year of residency. There have been many studies in which deep learning algorithms showed high diagnostic performance at the basic research level [14]. However, they did not provide comparisons with health-care professionals (i.e., human vs. machine), and few of the studies reported comparisons with healthcare professionals using the same test dataset. As shown in Table 4, in previous studies on deep learning algorithms for hip fractures, few assessments were made as to how deep learning algorithms affect clinicians’ diagnostic abilities [17–22]. Our study showed that the CAD system would be useful for aiding residents in the diagnosis of hip fracture.

The present study was associated with several limitations. First, the present dataset included cases of pathological fractures caused by metastatic bone tumors but did not include cases of osteomyelitis without fracture. It is desirable to consult a specialist as soon as possible in such cases; however, the CAD system developed in this study may not be able to point this out. Second, the image needs to be divided by preprocessing. A CAD system that can diagnose hip fractures without preprocessing from X-rays of both hips should be developed using the deep learning algorithm obtained in this study. Third, the diagnostic imaging test was not conducted in an actual clinical setting. This study was retrospective study conducted via a PACS-like web interface used by clinicians for medical imaging. Unlike the high-resolution monitors used in clinical practice, the reading of the images is done on a home personal computer, and therefore the diagnostic rate for clinicians may be underestimated. It is also possible that the incidence of “with fracture” images in clinical practice is different from the frequency of diagnosis in clinical practice. In this regard, future prospective studies in actual clinical settings using an actual PACS system are needed. Fourth, we have not been able to assess whether clinicians have fundamentally improved their diagnostic abilities in the diagnostic imaging test. In the diagnostic imaging test, clinicians read images without diagnostic aid and images with diagnostic aid consecutively. Because clinicians didn’t read the images at regular time intervals, the effect in terms of education is not known. In addition, the correctness criteria for the diagnostic imaging test was whether the clinician was able to answer the fracture site correctly on the basis of the presence or absence of a fracture. Grad-CAM presented the heat map as an indication of the fracture site, but it is unclear how much the heat map contributed to the clinician’s ability to read it.

## Conclusion

We developed a newly CAD system for the diagnosis of hip fracture based on a deep learning algorithm. This system provided high accuracy, sensitivity, and specificity. The areas activated on the heat map all corresponded to the areas pointed out by the orthopedic surgeon. The accuracy, sensitivity, and specificity of residents in the diagnosis of hip fracture improved when they used this CAD system. This system may aid residents in the diagnosis of hip fractures.

## Supplementary Information

**Additional file 1 **: **Supplemental methods**. **Supplemental Figure 1**. Image preprocessing. **Supplemental Figure 2**. Configuration diagram of the EfficientNet-B4 model. **Supplemental Figure 3**. The machine learning process. **Supplemental Figure 4**. The diagnostic test for clinicians. **Supplemental Figure 5**. Validation of the accuracy of heat maps generated by Grad-CAM [34, 35].

## Data Availability

The datasets analyzed during the current study available from the corresponding author on reasonable request.

## Abbreviations

AI: Artificial Intelligence
AUC: Area Under the Curve
CAD: Computer-Aided Diagnosis
CI: Confidence Interval
CT: Computed Tomography
DCNN: Deep Convolutional Neural Network
DICOM: Digital Imaging and COmmunications in Medicine
Grad-CAM: Gradient-weighted Class Activation Napping
MRI: Magnetic Resonance Imaging
PACS: Picture Archiving and Communication Systems
ROC: Receiver Operating Characteristic
