# Supplementary material for: Artificial intelligence improves the accuracy of residents in the diagnosis of hip fractures: a multicenter study
Source: BMC Musculoskelet Disord. 2021 May 3;22:407. doi: 10.1186/s12891-021-04260-2 (PMC8091525; doi:10.1186/s12891-021-04260-2)
Supplement: Supplementary file 1 — Additional file 1 : Supplemental methods. Supplemental Figure 1. Image preprocessing. Supplemental Figure 2. Configuration diagram of the EfficientNet-B4 model. Supplemental Figure 3. The machine learning process. Supplemental Figure 4. The diagnostic test for clinicians. Supplemental Figure 5. Validation of the accuracy of heat maps generated by Grad-CAM [34, 35]. [file 12891_2021_4260_MOESM1_ESM.docx]

Supplemental data

Supplemental methods

We used an Intel Core i7 8700 K, Ubuntu 18.04, and Python 3.7 to perform image processing on the target image data and train the algorithm.

We used Python 3.7 to train an algorithm for the analysis, and Pytorch 1.3 and Fast.ai 1.0 as deep learning libraries. We also used Nvidia's RTX 2070 GPU for learning and reasoning of deep learning. To perform transfer learning. We used the EfficientNet-B4 model, which was a pre-trained ImageNet model (Supplementary Figure 2). A deep convolutional neural network (DCNN) approach was used for the learning. The model was trained for two-class classification, with images with fractures as positive and images without fractures as negative.

Supplemental Figure 1. Image preprocessing


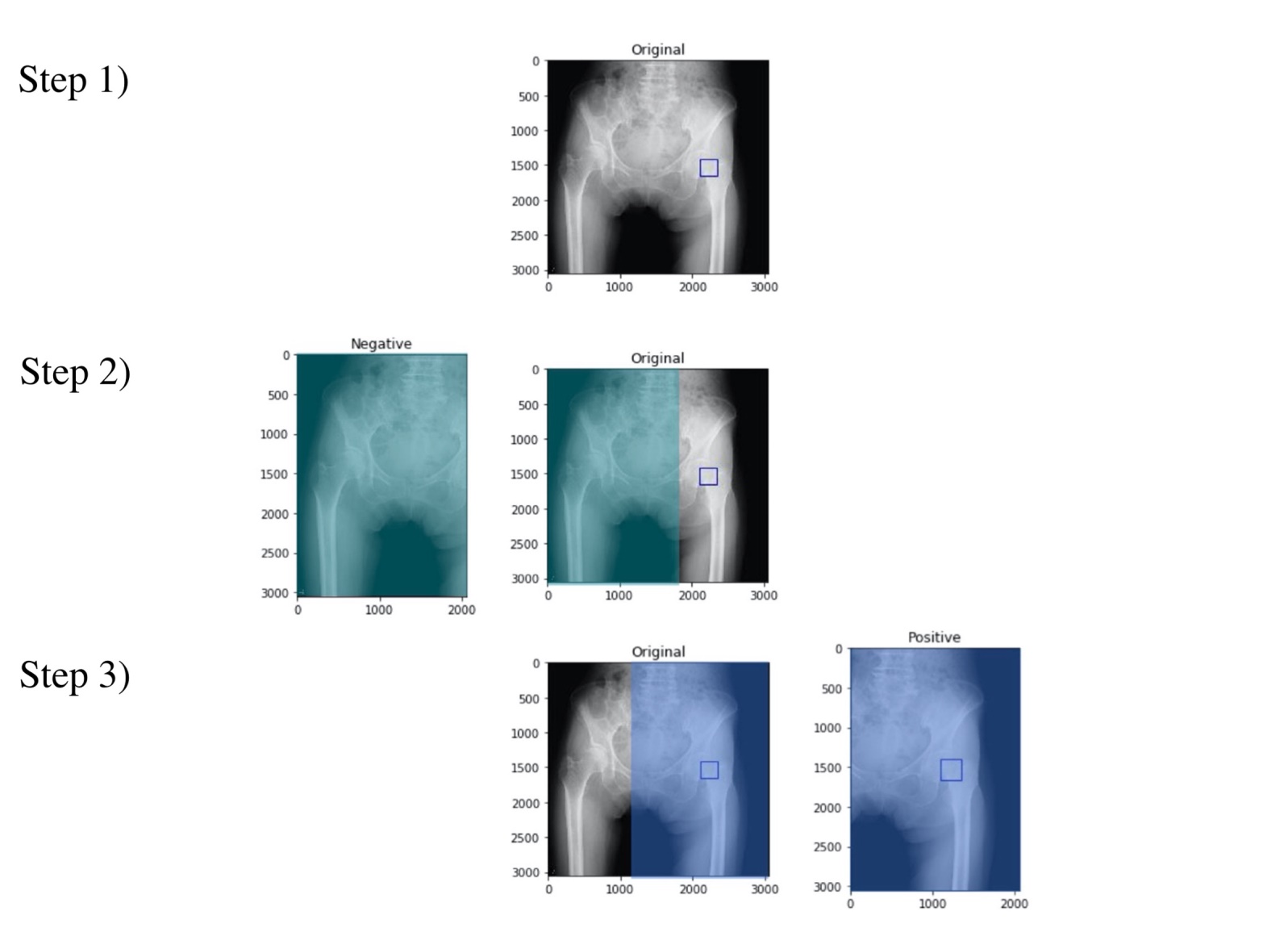


Step 1) For all 5242 plain X-rays, a rectangle of the shape including the fracture area was assigned by orthopedic surgeons.

Step 2) A margin of 50 pixels from the rectangle was set up and a dividing line was inserted; the image without the rectangle was used as the non-fractured image.

Step 3) The image of the fractured side was the same size as that of the non-fractured side and included a rectangle.

Supplemental Figure 2. Configuration diagram of the EfficientNet-B4 model


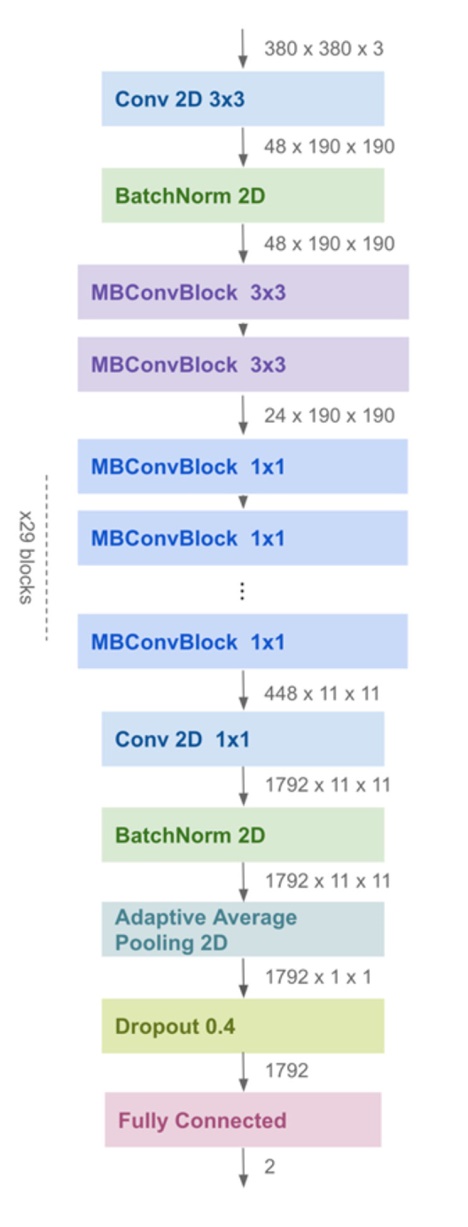


The EfficientNet-B4 model used in this study combines the depth, breadth, and input resolution of neural networks with the best efficiency to performance ratio in existing studies. It was adopted because the number of parameters was small in comparison to other learning models. The model was simple, and was suitable for transferring learning.

Supplemental Figure 3. The machine learning process


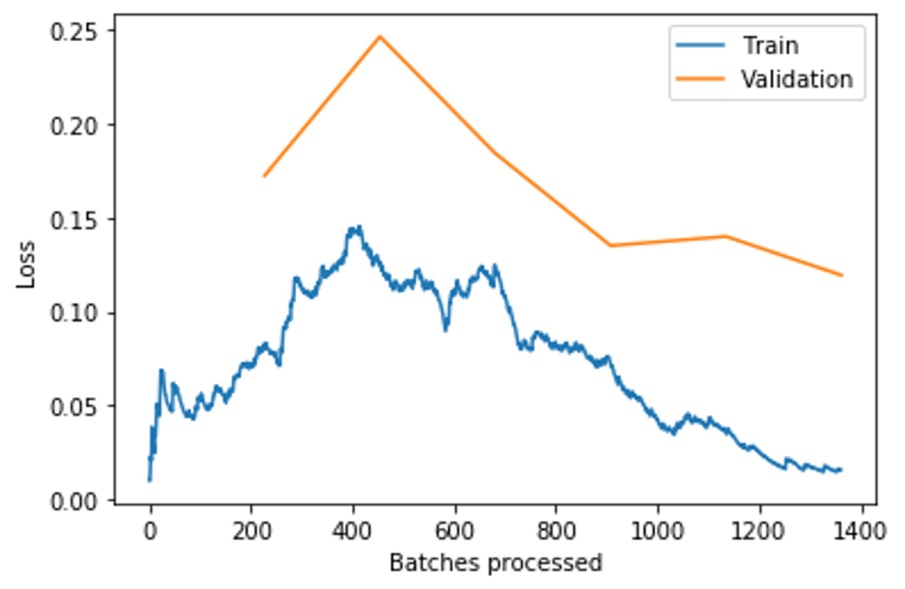


The training dataset and the validation dataset were used for training. The initial learning coefficients were increased from 0 to WarmStartup, then to about 10e-3 to 1 cycle, and then to Decay. The learning time was approximately 10 minutes per epoch, and the overall time was approximately one hour in six epochs. We used Adam as an optimizer. The batch size was set at 40, and the validation loss curve was confirmed in about 1200 batches, and it was judged that the plateau in performance was reached. Annealing of the LR was planned and the learning rate decay was performed in one cycle. In addition to the use of Dropout (p=0.4) in the EfficientNet-B4 model, which is included in the model adopted as a countermeasure against overlearning, we performed random mirroring on the vertical axis as data augmentation and light and dark changes randomly during learning. Early stopping is not used because of LR decay.

Supplemental Figure 4. The diagnostic test for clinicians


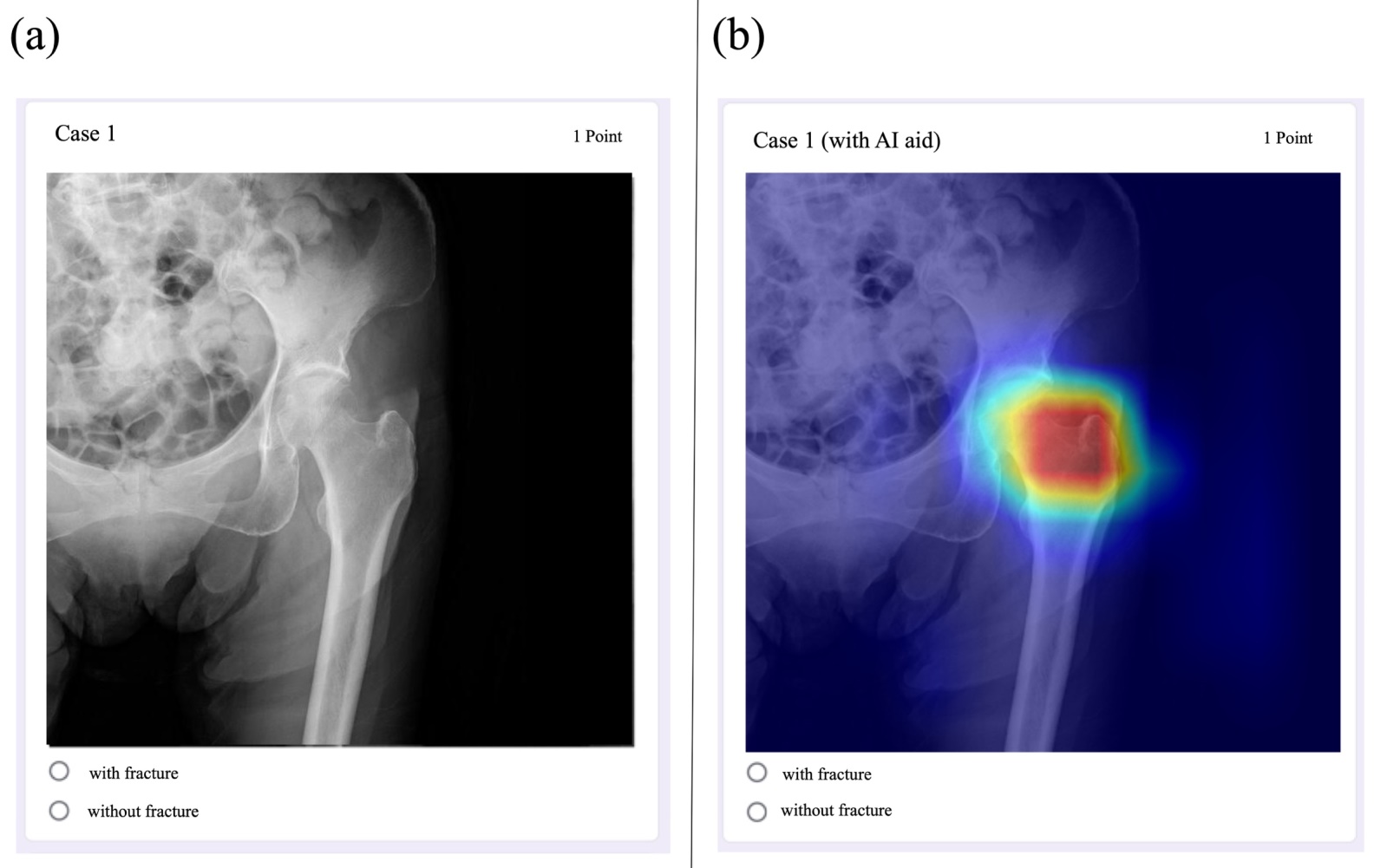


a) At first, the clinicians diagnosed the presence or absence of fracture by themselves

b) After the clinician answered, the CAD system added the visualization of the fracture to the same image. As a second test, the clinician responded again based on the hint.

Supplemental Figure 5. Validation of the accuracy of heat maps generated by Grad-CAM


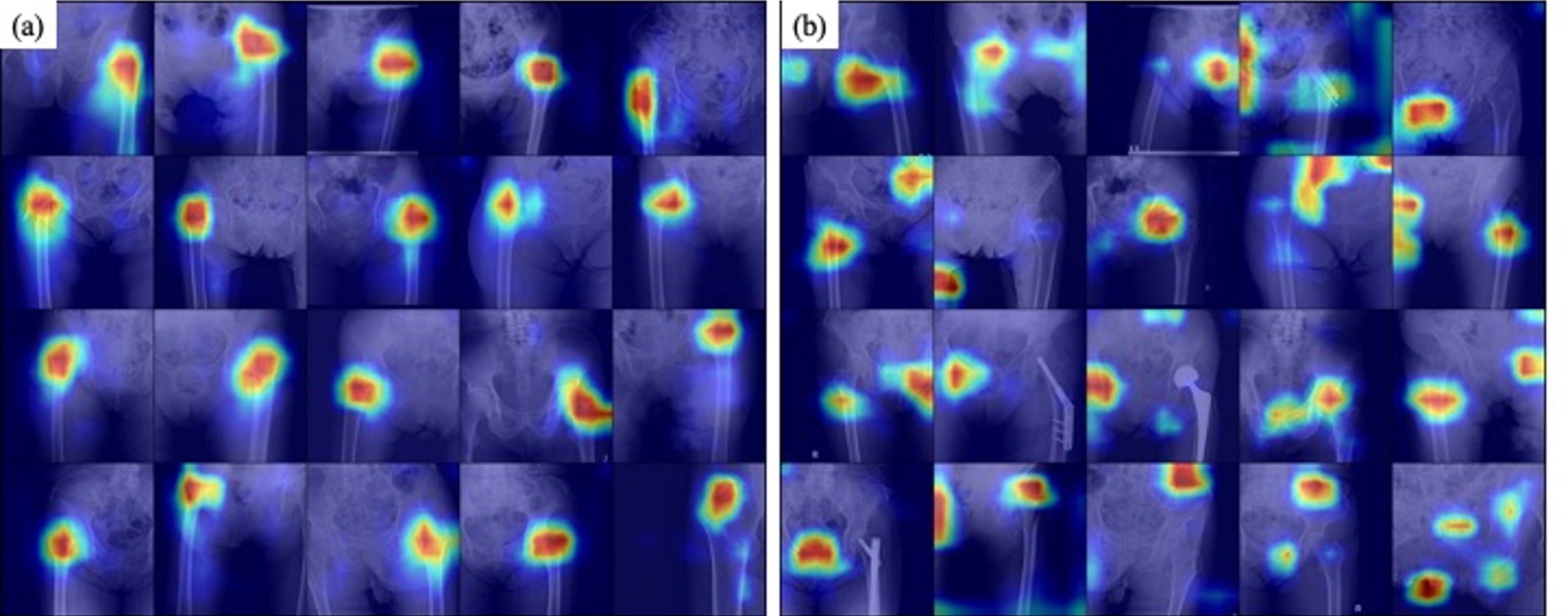


For the 20 "with Fracture" images, all 20 images had the same high-signal region on the heat map as the fracture site. On the other hand, 19 of the 20 "no fracture" images had high signal areas except from the femoral head to just above the trochanter in the 19 images, but one image had a high signal area in the greater trochanter.
